# Nondeterministic Syntactic Complexity

**DOI:** 10.1007/978-3-030-71995-1_23

**Published:** 2021-03-23

**Authors:** Robert S. R. Myers, Stefan Milius, Henning Urbat

**Affiliations:** 8 University of Oxford, Oxford, UK; 9 Sorbonne Université - LIP6, Paris, France; Friedrich-Alexander-Universität Erlangen-Nürnberg, Erlangen, Germany

## Abstract

We introduce a new measure on regular languages: their *nondeterministic syntactic complexity*. It is the least degree of any extension of the ‘canonical boolean representation’ of the syntactic monoid. Equivalently, it is the least number of states of any *subatomic* nondeterministic acceptor. It turns out that essentially all previous structural work on nondeterministic state-minimality computes this measure. Our approach rests on an algebraic interpretation of nondeterministic finite automata as deterministic finite automata endowed with semilattice structure. Crucially, the latter form a self-dual category.

## 1 Introduction

Regular languages admit a plethora of equivalent representations: finite automata, finite monoids, regular expressions, formulas of monadic second-order logic, and numerous others. In many cases, the most succinct representation is given by a *nondeterministic finite automaton (nfa).* Therefore, the investigation of state-minimal nfas is of both computational and mathematical interest. However, this turns out to be surprisingly intricate; in fact, the task of minimizing an nfa, or even of deciding whether a given nfa is minimal, is known to be PSPACE-complete [23]. One intuitive reason is that minimal nfas lack structure: a language may have many non-isomorphic minimal nondeterministic acceptors, and there are no clearly identified and easily verifiable mathematical properties distinguishing them from non-minimal ones. As a consequence, all known algorithms for nfa minimization (and related problems such as inclusion or universality testing) require some form of exhaustive search [9, 11, 26]. This sharply contrasts the situation for minimal *deterministic finite automata (dfa)*: they can be characterized by a universal property making them unique up to isomorphism, which immediately leads to efficient minimization.

In the present paper, we work towards the goal of bringing more structure into the theory of nondeterministic state-minimality. To this end, we propose a novel algebraic perspective on nfas resting on *boolean representations* of monoids, i.e. morphisms $M \to \mathbf{JSL}(S, S)$ from a monoid $M$ into the endomorphism monoid

⋆ Supported by Deutsche Forschungsgemeinschaft (DFG) under projects MI 717/5-2 and MI 717/7-1, and as part of the Research and Training Group 2475 "Cybercrime and Forensic Computing" (393541319/GRK2475/1-2019)

⋆⋆ Supported by Deutsche Forschungsgemeinschaft (DFG) under proj. SCHR 1118/8-2

S. Kiefer and C. Tasson (Eds.): FOSSACS 2021, LNCS 12650, pp. 448–468, 2021.
https://doi.org/10.1007/978-3-030-71995-1_23

of a finite join-semilattice $S$. Our focus lies on quotient monoids of the free monoid $\Sigma^*$ recognizing a given regular language $L \subseteq \Sigma^*$. The largest such monoid is $\Sigma^*$ itself, while the smallest one is the *syntactic monoid* $\mathsf{syn}(L)$. For both of them, $L$ induces a *canonical boolean representation*

$$\Sigma^* \to \mathbf{JSL}(\mathsf{SLD}(L), \mathsf{SLD}(L) \qquad \text{and} \qquad \mathsf{syn}(L) \to \mathbf{JSL}(\mathsf{SLD}(L), \mathsf{SLD}(L))$$

on the semilattice $\mathsf{SLD}(L)$ of all finite unions of left derivatives of $L$. The first representation gives rise to an algebraic characterization of minimal nfas:

**Theorem.** The size of a state-minimal nfa for $L$ equals the least degree of any extension of the canonical representation of $\Sigma^*$ induced by $L$.

Here, the *degree* of a representation refers to the number of join-irreducibles of the underlying semilattice. In the light of this result, it is natural to ask for an analogous automata-theoretic perspective on the canonical representation of $\mathsf{syn}(L)$ and its extensions. For this purpose, we introduce the class of *subatomic* nfas, a generalization of *atomic* nfas earlier introduced by Brzozowski and Tamm [6]. In order to get a handle on them, we employ an algebraic framework that interprets nfas in terms of **JSL**-*dfas*, i.e. deterministic finite automata in the category of semilattices. In this setting, the semilattice $\mathsf{SLD}(L)$ used in the canonical representations naturally arises as the *minimal* **JSL**-dfa for the language $L$. We shall demonstrate that much of the structure theory of (sub-)atomic nfas reduces to the observation that the category of **JSL**-dfas is *self-dual*. Our main result gives an algebraic characterization of minimal subatomic nfas:

**Theorem.** The size of a state-minimal subatomic nfa for $L$ equals the least degree of any extension of the canonical representation of $\mathsf{syn}(L)$.

We call the measure suggested by the above theorem the *nondeterministic syntactic complexity* of the language $L$. It turns out to be extremely natural: as illustrated in Section 5, essentially all existing work on the structure of state-minimal nfas implicitly identifies classes of languages whose nondeterministic state complexity equals their nondeterministic syntactic complexity, and thus is actually concerned with computing minimal subatomic acceptors.

## 2 Preliminaries

We start by introducing some notation and terminology used in the paper.

*Semilattices.* A *(join-)semilattice* is a poset $(S, \leq_S)$ in which every finite subset $X \subseteq S$ has a least upper bound, a.k.a. join, denoted by $\bigvee X$. A *morphism* of semilattices is a map preserving all finite joins. Let **JSL** denote the category of join-semilattices and their morphisms. An element $j$ of a semilattice $S$ is *join-irreducible* if for all finite subsets $X \subseteq S$ with $j = \bigvee X$ one has $j \in X$. Let

$$J(S) = \{\, j \in S \;:\; j \text{ is join-irreducible} \,\}.$$

Let $2 = \{0, 1\}$ denote the two-element semilattice with $0 \leq 1$. Since $2 \cong (\mathcal{P}(1), \subseteq)$ is the free semilattice on a single generator, morphisms from 2 into a semilattice $S$

correspond uniquely to elements of $S$. Similarly, a morphism $f\colon S \to 2$ corresponds uniquely to a *prime filter* $F = f^{-1}[1] \subseteq S$, i.e. an upwards closed subset such that $\bigvee X \in F$ implies $X \cap F \neq \emptyset$ for every finite subset $X \subseteq S$. If $S$ is finite, prime filters are precisely the sets $F = \{s \in S : s \not\leq s_0\}$ for $s_0 \in S$. If $S$ is a subsemilattice of a semilattice $T$, every prime filter $F$ of $S$ can be extended to the prime filter $T \setminus (\downarrow(S \setminus F))$ of $T$, where $\downarrow X = \{\, t \in T \;:\; t \leq x \text{ for some } x \in X \,\}$ denotes the down-closure of a subset $X \subseteq T$. Equivalently, every morphism $f\colon S \to 2$ can be extended to a morphism $g\colon T \to 2$. In category-theoretic terminology, this means that the semilattice 2 forms an injective object of **JSL**.

The category $\mathbf{JSL}_\mathsf{f}$ of finite semilattices is *self-dual* [25]. The equivalence functor $\mathbf{JSL}_\mathsf{f} \xrightarrow{\simeq} \mathbf{JSL}_\mathsf{f}^{\mathsf{op}}$ sends a semilattice $S$ to its *dual semilattice* $S^{\mathsf{op}}$ obtained by reversing the order, and a morphism $f\colon S \to T$ to the morphism $f^*\colon T^{\mathsf{op}} \to S^{\mathsf{op}}$ mapping $t \in T$ to the $\leq_S$-largest element $s \in S$ with $f(s) \leq_T t$. Note that $f$ is *adjoint* to $f^*$: for $s \in S$ and $t \in T$ we have $f(s) \leq_T t$ iff $s \leq_S f^*(t)$.

*Languages.* A *language* is a subset $L$ of $\Sigma^*$, the set of finite words over an alphabet $\Sigma$. We let $\overline{L} = \Sigma^* \setminus L$ denote the *complement* and $L^\mathsf{r} = \{w^\mathsf{r} : w \in L\}$ the *reverse*, where $w^\mathsf{r} = a_n \dots a_1$ for $w = a_1 \dots a_n$. The *left derivatives*, *right derivatives* and *two-sided derivatives* of $L$ are, respectively, given by $u^{-1}L = \{w \in \Sigma^* : uw \in L\}$, $Lv^{-1} = \{w \in \Sigma^* : wv \in L\}$ and $u^{-1}Lv^{-1} = \{w \in \Sigma^* : uwv \in L\}$ for $u, v \in \Sigma^*$. More generally, for $U \subseteq \Sigma^*$ the language $U^{-1}L = \bigcup_{u \in U} u^{-1}L$ is called the *left quotient* of $L$ w.r.t. $U$. We define the following sets of languages generated by $L$:

- $\mathsf{LD}(L) = \{u^{-1}L : u \in \Sigma^*\}$, the set of all left derivatives of $L$;
- $\mathsf{SLD}(L)$, its closure under finite union;
- $\mathsf{BLD}(L)$, its closure under all set-theoretic boolean operations;
- $\mathsf{BLRD}(L)$, its closure under all boolean operations and right derivatives.

In other words, $\mathsf{SLD}(L)$ is the $\cup$-semilattice of all left quotients of $L$, or equivalently, the $\cup$-subsemilattice of $\mathcal{P}(\Sigma^*)$ generated by all left derivatives. Moreover, $\mathsf{BLD}(L)$ and $\mathsf{BLRD}(L)$ form the boolean subalgebras of $\mathcal{P}(\Sigma^*)$ generated by all left derivatives and all two-sided derivatives, respectively.

## 3 Duality Theory of Semilattice Automata

In this section, we set up the algebraic framework in which nondeterministic automata can be studied. Since it involves considering several different types of automata, it is convenient to view them all as instances of a general categorical concept. For the rest of this paper, let $\Sigma$ denote a fixed finite input alphabet.

**Definition 3.1.** Let $\mathscr{C}$ be a category and let $X, Y \in \mathscr{C}$ be two fixed objects. An *automaton* in $\mathscr{C}$ is a quadruple $(S, \delta, i, f)$ consisting of an object $S \in \mathscr{C}$ of *states*, a family $\delta = (\delta_a\colon S \to S)_{a \in \Sigma}$ of morphisms representing *transitions*, and two morphisms $i\colon X \to S$ and $f\colon S \to Y$ representing *initial* and *final* states (see the left-hand diagram below). A *morphism* between automata $(S, \delta, i, f)$ and $(S', \delta', i', f')$ is given by a morphism $h\colon S \to S'$ in $\mathscr{C}$ preserving transitions, initial states and final states, i.e. making the right-hand diagram below commute for all $a \in \Sigma$:

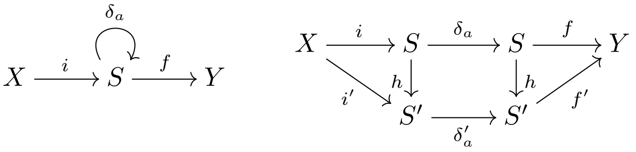

Let $\mathbf{Aut}(\mathscr{C})$ denote the category of automata in $\mathscr{C}$ and their morphisms.

**Notation 3.2.** We put $\delta_w := \delta_{a_n} \circ \cdots \circ \delta_{a_1}$ for $w = a_1 \ldots a_n$ in $\Sigma^*$.

**Example 3.3.** (1) An automaton $D = (S, \delta, i, f)$ in **Set**, the category of sets and functions, with $X = 1$ and $Y = 2$, is precisely a classical *deterministic automaton*. It is called a *dfa* if $S$ is finite. We identify the map $i: 1 \to S$ with an initial state $s_0 = i(*) \in S$, and the map $f: S \to 2$ with a set $F = f^{-1}[1] \subseteq S$ of final states. The language $L(D, s)$ *accepted* by a state $s \in S$ is the set of all words $w \in \Sigma^*$ such that $\delta_w(s) \in F$. The language $L(D)$ *accepted* by $D$ is the language accepted by the state $s_0$.

(2) An automaton $N = (S, \delta, i, f)$ in **Rel**, the category of sets and relations, with $X = Y = 1$, is precisely a classical *nondeterministic automaton*. It is called an *nfa* if $S$ is finite. We identify $i \subseteq 1 \times S$ with a set $I \subseteq S$ of initial states and $f \subseteq S \times 1$ with a set $F \subseteq S$ of final states. Thus, in our view an nfa may have multiple initial states. The language $L(N, R)$ *accepted* by a subset $R \subseteq S$ consists of all $w \in \Sigma^*$ such that $(r, s) \in \delta_w$ for some $r \in R$ and $s \in F$. The language $L(N)$ *accepted* by $N$ is the language accepted by the set $I$.

(3) An automaton $A = (S, \delta, i, f)$ in **JSL** with $X = Y = 2$, shortly a **JSL**-*automaton*, is given by a semilattice $S$ of states, a family $\delta = (\delta_a : S \to S)_{a \in \Sigma}$ of semilattice morphisms specifying transitions, an initial state $s_0 \in S$ (corresponding to $i: 2 \to S$), and a prime filter $F \subseteq S$ of final states (corresponding to $f: S \to 2$). It is called a **JSL**-*dfa* if $S$ is finite. The language *accepted* by a state $s \in S$ or by the automaton $A$, resp., is defined as for deterministic automata.

**Remark 3.4 (JSL-dfas vs. nfas).** Dfas, nfas and **JSL**-dfas are expressively equivalent; they all accept precisely the regular languages. The interest of **JSL**-dfas is that they constitute an algebraic representation of nfas:

(1) Every **JSL**-dfa $A = (S, \delta, s_0, F)$ induces an equivalent nfa $J(A)$ on the set $J(S)$ of join-irreducibles of $S$. Given $s, t \in J(S)$ and $a \in \Sigma$, there is a transition $s \xrightarrow{a} t$ in $J(A)$ iff $t \leq \delta_a(s)$; the initial states are those $s \in J(S)$ with $s \leq s_0$, and the final states form the set $J(S) \cap F$.

(2) Conversely, for every nfa $N = (Q, \delta, I, F)$, the *subset construction* yields an equivalent **JSL**-dfa $\mathcal{P}(N)$ with states $\mathcal{P}(Q)$ (the $\cup$-semilattice of subsets of $Q$), transitions $\mathcal{P}\delta_a : \mathcal{P}(Q) \to \mathcal{P}(Q)$, $X \mapsto \delta_a[X]$, initial state $I \in \mathcal{P}(Q)$, and final states those subsets of $Q$ containing some state from $F$. Note that $J(\mathcal{P}(Q)) \cong Q$.

It follows that the task of finding a state-minimal nfa for a given language is equivalent to finding a **JSL**-dfa with a minimum number of join-irreducibles [4]. This idea has recently been extended to a general coalgebraic framework [32, 39].

Recall that the *minimal dfa* [7] for a regular language $L$, denoted by $\mathsf{dfa}(L)$, has states $\mathsf{LD}(L)$ (the set of left derivatives of $L$), transitions $K \xrightarrow{a} a^{-1}K$ for $K \in \mathsf{LD}(L)$ and $a \in \Sigma$, initial state $L = \varepsilon^{-1}L$, and final states those $K \in \mathsf{LD}(L)$ containing $\varepsilon$. Up to isomorphism, it can be characterized as the unique dfa accepting $L$ that is *reachable* (i.e. every state is reachable from the initial state via transitions) and *simple* (i.e. any two distinct states accept distinct languages). We now develop the analogous concepts for **JSL**-automata; they are instances of the categorical theory of minimality due to Arbib and Manes [3] and Goguen [15]. Let us first observe that every language has two canonical infinite **JSL**-acceptors:

**Definition 3.5.** Let $L \subseteq \Sigma^*$ be a language.

(1) The *initial* **JSL**-*automaton* $\mathsf{Init}(L)$ for $L$ has states $\mathcal{P}_{\mathsf{f}}(\Sigma^*)$ (the $\cup$-semilattice of finite subsets of $\Sigma^*$), initial state $\{\varepsilon\}$, final states all $X \in \mathcal{P}_{\mathsf{f}}(\Sigma^*)$ with $X \cap L \neq \emptyset$, and transitions $X \mapsto Xa = \{xa \ : \ x \in X\}$ for $X \in \mathcal{P}_{\mathsf{f}}(\Sigma^*)$ and $a \in \Sigma$.

(2) The *final* **JSL**-*automaton* $\mathsf{Fin}(L)$ for $L$ has states $\mathcal{P}(\Sigma^*)$ (the $\cup$-semilattice of all languages), initial state $L$, final states all languages $K$ containing $\varepsilon$, and transitions $K \mapsto a^{-1}K$ for $K \in \mathcal{P}(\Sigma^*)$ and $a \in \Sigma$.

As suggested by the terminology, these automata form the initial and the final object in the category of **JSL**-automata accepting $L$:

**Lemma 3.6 [3,15].** *For every* **JSL**-*automaton* $A = (S, \delta, s_0, F)$ *accepting the language* $L \subseteq \Sigma^*$*, there exist unique* **JSL**-*automata morphisms*

$$e_A\colon \mathsf{Init}(L) \to A \qquad \text{and} \qquad m_A\colon A \to \mathsf{Fin}(L).$$

*The map* $e_A$ *sends* $\{w_1, \ldots, w_n\} \in \mathcal{P}_{\mathsf{f}}(\Sigma^*)$ *to the state* $\bigvee_{i=1}^n \delta_{w_i}(s_0)$*, and the map* $m_A$ *sends a state* $s \in S$ *to* $L(A, s)$*, the language accepted by* $s$*.*

**Definition 3.7.** A **JSL**-automaton $A = (S, \delta, s_0, F)$ is called

(1) *reachable* if the unique morphism $e_A\colon \mathsf{Init}(L) \to A$ is surjective, i.e. every state is of the form $\bigvee_{i=1}^n \delta_{w_i}(s_0)$ for some $w_1, \ldots, w_n \in \Sigma^*$;

(2) *simple* if the unique morphism $m_A\colon A \to \mathsf{Fin}(L)$ in injective, i.e. any two distinct states accept distinct languages;

(3) *minimal* if it is both reachable and simple.

**Remark 3.8.** (1) The category **Aut**(**JSL**) has a factorization system given by surjective and injective morphisms. Thus, for every **JSL**-automata morphism $h\colon (S, \delta, i, f) \to (S', \delta', i', f')$ with image factorization $h = (S \overset{e}{\twoheadrightarrow} S'' \overset{m}{\rightarrowtail} S')$ in **JSL**, there exists a unique **JSL**-automaton structure $(S'', \delta'', i'', f'')$ on $S''$ making both $e$ and $m$ automata morphisms. We call $e$ the *coimage* and $m$ the *image* of $h$. *Subautomata* and *quotient automata* of **JSL**-automata are represented by injective and surjective morphisms, respectively.

(2) Every **JSL**-automaton $A$ has a unique reachable subautomaton $\mathsf{reach}(A) \rightarrowtail A$, the *reachable part* of $A$. It is the smallest subautomaton of $A$ and arises as the image of the unique morphism $e_A\colon \mathsf{Init}(L) \to A$. Thus,

$$A \text{ is reachable} \quad \text{iff} \quad A \cong \mathsf{reach}(A) \quad \text{iff} \quad A \text{ has no proper subautomaton.}$$

Let us emphasize that a state in $\mathsf{reach}(A)$ is not necessarily reachable when $A$ is viewed as an ordinary dfa. For distinction, we thus call a state **JSL**-*reachable* if it lies in $\mathsf{reach}(A)$, and *dfa-reachable* if it is reachable in the usual sense.

(3) Dually, every **JSL**-automaton $A$ has a unique simple quotient automaton $A \twoheadrightarrow \mathsf{simple}(A)$, the *simplification* of $A$. It is the smallest quotient automaton of $A$ and arises as the coimage of the unique morphism $m_A\colon A \to \mathsf{Fin}(L)$. Thus,

$$A \text{ is simple} \quad \text{iff} \quad A \cong \mathsf{simple}(A) \quad \text{iff} \quad A \text{ has no proper quotient automaton.}$$

(4) Every language $L \subseteq \Sigma^*$ has a minimal **JSL**-automaton, unique up to isomorphism. It can be constructed as the image of the unique automata morphism $h_L\colon \mathsf{Init}(L) \to \mathsf{Fin}(L)$. Since $h_L$ sends $\{w_1, \ldots, w_n\} \in \mathcal{P}_\mathsf{f}(\Sigma^*)$ to the language $\bigcup_{i=1}^n w_i^{-1}L$, the minimal automaton of $L$ is the subautomaton $\mathsf{SLD}(L)$ of $\mathsf{Fin}(L)$ carried by the semilattice of finite unions of left derivatives of $L$.

**Example 3.9.** The minimal **JSL**-dfa accepting $L = \{a, aa\}$ is shown below, with the dashed lines representing the partial order.

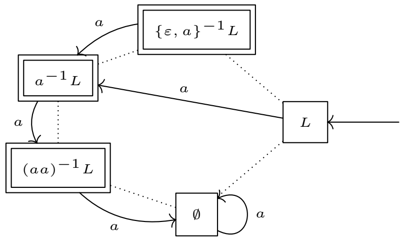

**Remark 3.10.** The self-duality of $\mathbf{JSL}_\mathsf{f}$ lifts to a self-duality of the category of **JSL**-dfas. The equivalence functor $\mathbf{Aut}(\mathbf{JSL}_\mathsf{f}) \xrightarrow{\simeq} \mathbf{Aut}(\mathbf{JSL}_\mathsf{f})^\mathsf{op}$ maps a **JSL**-dfa $A = (S, (\delta_a\colon S \to S)_{a\in\Sigma}, i\colon 2 \to S, f\colon S \to 2)$ to its *dual automaton*

$$A^\mathsf{op} = (S^\mathsf{op}, (\delta_a^*\colon S^\mathsf{op} \to S^\mathsf{op})_{a\in\Sigma}, f^*\colon 2 \to S^\mathsf{op}, i^*\colon S^\mathsf{op} \to 2),$$

using that $2^\mathsf{op} \cong 2$. Thus, the initial state of $A^\mathsf{op}$ is the $\leq_S$-largest non-final state of $A$, and its final states are those $s \in S$ with $s_0 \not\leq_S s$. Given $s, t \in S$ and $a \in \Sigma$, there is a transition $s \xrightarrow{a} t$ in $A^\mathsf{op}$ iff $t$ is the $\leq_S$-largest state with $\delta_a(t) \leq_S s$.

The dualization of **JSL**-dfas can be seen as an algebraic generalization of the reversal operation on nfas. Recall that the *reverse* of an nfa $N$ is the nfa $N^\mathsf{r}$ obtained by flipping all transitions and swapping initial and final states. If $N$ accepts the language $L$, then $N^\mathsf{r}$ accepts the reverse language $L^\mathsf{r}$.

**Lemma 3.11.** *For each nfa $N = (Q, \delta, I, F)$, we have the* **JSL**-*dfa isomorphism*

$$[\mathcal{P}(N)]^\mathsf{op} \xrightarrow{\cong} \mathcal{P}(N^\mathsf{r}), \qquad X \mapsto \overline{X} = Q \setminus X.$$

The following lemma summarizes some important properties of $A^{\mathsf{op}}$:

**Lemma 3.12.** *Let $A = (S, \delta, i, f)$ be a* **JSL***-dfa.*

(1) *For every $s \in S$, we have $L(A^{\mathsf{op}}, s) = \{\, w \in \Sigma^* \;:\; \delta_{w^{\mathsf{r}}}(s_0) \not\leq_S s \,\}$.*

(2) *If $A$ accepts the language $L$, then $A^{\mathsf{op}}$ accepts the reverse language $L^{\mathsf{r}}$.*

(3) *We have $[\mathsf{reach}(A)]^{\mathsf{op}} \cong \mathsf{simple}(A^{\mathsf{op}})$. Thus, $A$ is reachable iff $A^{\mathsf{op}}$ is simple.*

Our next goal is to give, for every regular language $L$, dual characterizations of $\mathsf{SLD}(L)$, $\mathsf{BLD}(L)$ and $\mathsf{BLRD}(L)$, the **JSL**-subautomata of $\mathsf{Fin}(L)$ carried by all finite unions of left derivatives, boolean combinations of left derivatives and boolean combinations of two-sided derivatives, respectively. These results form the core of our duality-based approach to (sub-)atomic nfas in the next section. The minimal **JSL**-dfa $\mathsf{SLD}(L)$ admits the following dual description:

**Proposition 3.13.** *For every regular language $L$, the minimal* **JSL***-dfas for $L$ and $L^{\mathsf{r}}$ are dual. More precisely, we have the* **JSL***-dfa isomorphism*

$$\mathrm{dr}_L \colon [\mathsf{SLD}(L^{\mathsf{r}})]^{\mathsf{op}} \xrightarrow{\cong} \mathsf{SLD}(L), \qquad K \mapsto (\overline{K^{\mathsf{r}}})^{-1}L.$$

**Remark 3.14.** (1) The isomorphism $\mathrm{dr}_L$ induces a bijection between the *left* and *right factors* of $L$, i.e. the inclusion-maximal left/right solutions of $X \cdot Y \subseteq L$. Conway [10] observed that the left and right factors are respectively $\{\overline{K^{\mathsf{r}}} : K \in \mathsf{SLD}(L^{\mathsf{r}})\}$ and $\{\overline{K} : K \in \mathsf{SLD}(L)\}$ and that they biject. Backhouse [5] observed that they are dually isomorphic posets. Proposition 3.13 provides an explicit automata-theoretic lattice isomorphism arising canonically via duality.

(2) The isomorphism $\mathrm{dr}_L$ is tightly connected to the *dependency relation* [18, 20] of a regular language $L$, i.e. the binary relation given by

$$\mathcal{DR}_L \subseteq \mathsf{LD}(L) \times \mathsf{LD}(L^r), \qquad \mathcal{DR}_L(u^{-1}L, v^{-1}L^r) :\iff uv^r \in L.$$

Its restriction $\mathcal{DR}_L^j := \mathcal{DR}_L \cap J(\mathsf{SLD}(L)) \times J(\mathsf{SLD}(L^r))$ to the $\cup$-irreducible left derivatives of $L$ and $L^{\mathsf{r}}$ is called the *reduced dependency relation.* The following theorem shows that the semilattice of left quotients and the dependency relation are essentially the same concepts. In part (3), we use that the isomorphism $\mathrm{dr}_L$ restricts to a bijection between the $\cup$-irreducible derivatives of $L^{\mathsf{r}}$ and the meet-irreducible elements of the lattice $\mathsf{SLD}(L)$.

**Theorem 3.15 (Dependency theorem).**

(1) *We have the* **JSL***-isomorphism*

$$\mathsf{SLD}(L) \xrightarrow{\cong} (\{\mathcal{DR}_L[X] : X \subseteq \mathsf{LD}(L)\}, \cup, \emptyset), \qquad K \mapsto \{v^{-1}L^{\mathsf{r}} : v \in K^{\mathsf{r}}\}.$$

*Note that its codomain forms a subsemilattice of $\mathcal{P}(\mathsf{LD}(L^{\mathsf{r}}))$.*

(2) *For all $u, v \in \Sigma^*$ we have $\mathcal{DR}_L(u^{-1}L, v^{-1}L^r) \iff u^{-1}L \not\subseteq \mathrm{dr}_L(v^{-1}L^r)$.*

(3) *The following diagram in* **Rel** *commutes:*

$$\begin{array}{ccc} J(\mathsf{SLD}(L^{\mathsf{r}})) & \xrightarrow[\cong]{\mathrm{dr}_L} & M(\mathsf{SLD}(L)) \\ {\scriptstyle \mathcal{DR}_L^j}\uparrow & & \uparrow{\scriptstyle \not\subseteq} \\ J(\mathsf{SLD}(L)) & = & J(\mathsf{SLD}(L)) \end{array}$$

Let us now turn to a dual characterization of the **JSL**-dfa $\mathsf{BLD}(L)$:

**Proposition 3.16.** *For every regular language $L$, the* **JSL**-*dfa* $\mathsf{BLD}(L)$ *is dual to the subset construction of the minimal dfa for* $L^{\mathsf{r}}$*:*

$$[\mathsf{BLD}(L)]^{\mathsf{op}} \;\cong\; \mathcal{P}(\mathsf{dfa}(L^{\mathsf{r}})).$$

*The isomorphism maps* $\{w_1^{-1}L^{\mathsf{r}}, \dots, w_n^{-1}L^{\mathsf{r}}\} \in \mathcal{P}(\mathsf{dfa}(L^{\mathsf{r}}))$ *to* $\bigcap_{i=1}^{n} \overline{\mathrm{At}(w_i^{\mathsf{r}})}$*, where* $\mathrm{At}(x)$ *is the unique atom (= join-irreducible) of* $\mathsf{BLD}(L)$ *containing* $x$*.*

To state the dual characterization of $\mathsf{BLRD}(L)$, we recall two standard concepts from algebraic language theory [33]. The *transition monoid* of a deterministic automaton $D = (S, \delta, i, f)$ is the image $\mathsf{tm}(D) \subseteq \mathbf{Set}(S, S)$ of the morphism

$$\Sigma^* \to \mathbf{Set}(S, S), \quad w \;\mapsto\; \delta_w.$$

Thus, $\mathsf{tm}(M)$ is carried by the set of extended transition maps $\delta_w$ ($w \in \Sigma^*$) with multiplication given by $\delta_v \bullet \delta_w = \delta_{vw}$ and unit $id_S = \delta_\varepsilon \colon S \to S$. We may view $\mathsf{tm}(D)$ as a deterministic automaton with initial state $id_S$, final states all $\delta_w$ such that $w$ is accepted by $D$, and transitions $\delta_w \xrightarrow{a} \delta_{wa}$ for $w \in \Sigma^*$ and $a \in \Sigma$. This automaton accepts the same language as $D$. The *syntactic monoid* $\mathsf{syn}(L)$ of a regular language $L \subseteq \Sigma^*$ is the transition monoid of its minimal dfa:

$$\mathsf{syn}(L) = \mathsf{tm}(\mathsf{dfa}(L)).$$

Equivalently, $\mathsf{syn}(L)$ is the quotient monoid of the free monoid $\Sigma^*$ modulo the *syntactic congruence* of $L$, i.e the monoid congruence on $\Sigma^*$ given by

$$v \equiv_L w \quad \text{iff} \quad \forall x, y \in \Sigma^* : xvy \in L \iff xwy \in L.$$

The associated surjective monoid morphism $\mu_L \colon \Sigma^* \twoheadrightarrow \mathsf{syn}(L)$, mapping $w \in \Sigma^*$ to its congruence class $[w]_L \in \mathsf{syn}(L)$, is called the *syntactic morphism.*

**Proposition 3.17.** *For every regular language $L$, the* **JSL**-*dfa* $\mathsf{BLRD}(L)$ *is dual to the subset construction of* $\mathsf{syn}(L^{\mathsf{r}})$*, viewed as a dfa:*

$$[\mathsf{BLRD}(L)]^{\mathsf{op}} \;\cong\; \mathcal{P}(\mathsf{syn}(L^{\mathsf{r}})).$$

*The isomorphism maps* $\{\, [w_1]_{L^{\mathsf{r}}}, \dots, [w_n]_{L^{\mathsf{r}}} \,\} \in \mathcal{P}(\mathsf{syn}(L^{\mathsf{r}}))$ *to* $\bigcap_{i=1}^{n} \overline{\mathrm{At}(w_i{}^{\mathsf{r}})}$*, with* $\mathrm{At}(x)$ *denoting the unique atom of* $\mathsf{BLRD}(L)$ *containing* $x$*.*

Our final duality result in this section concerns the *transition semiring* [35], a generalization of the transition monoid to **JSL**-automata. Note that the monoid $\mathbf{JSL}(S, S)$ of endomorphisms of a semilattice $S$ forms an idempotent semiring with join defined pointwise: for any $f, g \colon S \to S$, the morphism $f \vee g \colon S \to S$ is given by $s \mapsto f(s) \vee g(s)$. The transition semiring of a **JSL**-automaton $A = (S, \delta, i, f)$ is the image $\mathsf{ts}(A) \subseteq \mathbf{JSL}(S, S)$ of the semiring morphism

$$\mathcal{P}_{\mathsf{f}}(\Sigma^*) \to \mathbf{JSL}(S, S), \quad \{w_1, \dots, w_n\} \;\mapsto\; \bigvee_{i=1}^{n} \delta_{w_i}.$$

Here $\mathcal{P}_{\mathsf{f}}(\Sigma^*)$ is the free idempotent semiring on $\Sigma$, with composition given by concatenation of languages and join given by union. Thus, $\mathsf{ts}(A)$ is the semiring carried by all morphisms $\bigvee_{i=1}^{n} \delta_{w_i}$ for $w_1, \dots, w_n \in \Sigma^*$, with join given as above and multiplication $\bigvee_j \delta_{v_j} \bullet \bigvee_i \delta_{w_i} = \bigvee_{i,j} \delta_{v_j w_i}$. We view $\mathsf{ts}(A)$ as a **JSL**-automaton with initial state $id_S = \delta_\varepsilon$, final states all $\bigvee_i \delta_{w_i}$ such that some $w_i$ is accepted by $A$, and transitions $\bigvee_{i=1}^{n} \delta_{w_i} \xrightarrow{a} \bigvee_{i=1}^{n} \delta_{w_i a}$ for $w_1, \dots, w_n \in \Sigma^*$ and $a \in \Sigma$. This **JSL**-automaton is reachable and accepts the same language as $A$. It has the following dual characterization:

**Notation 3.18.** Given a simple **JSL**-automaton $A = (S, \delta, i, f)$, the subautomaton of $\mathsf{Fin}(L)$ obtained by closing $S$ (viewed as a set of languages) under right derivatives is called the *right-derivative closure* of $A$ and denoted $\mathsf{rdc}(A)$.

**Proposition 3.19.** *Let $A$ be a reachable* **JSL***-dfa. Then the transition semiring of $A$, viewed as a* **JSL***-dfa, is dual to the right-derivative closure of $A^{\mathsf{op}}$:*

$$[\mathsf{ts}(A)]^{\mathsf{op}} \cong \mathsf{rdc}(A^{\mathsf{op}}).$$

Note that both $[\mathsf{ts}(A)]^{\mathsf{op}}$ and $\mathsf{rdc}(A^{\mathsf{op}})$ are simple, hence subautomata of $\mathsf{Fin}(L)$. Thus, the isomorphism just expresses that their states accept the same languages.

## 4 Boolean Representations and Subatomic NFAs

Based upon the duality results of the previous section, we will now introduce our algebraic approach to nondeterministic state minimality. It rests on the concept of a representation of a monoid on a finite semilattice.

**Definition 4.1 (Boolean representation).** Let $M$ be a monoid.

(1) A *boolean representation* of $M$ is given by a finite semilattice $S$ together with a monoid morphism $\rho\colon M \to \mathbf{JSL}(S, S)$. The *degree* of $\rho$ is

$$\deg(\rho) := |J(S)|.$$

(2) Given boolean representations $\rho_i\colon M \to \mathbf{JSL}(S_i, S_i)$, $i = 1, 2$, an *equivariant map* $f\colon \rho_1 \to \rho_2$ is a **JSL**-morphism $f\colon S_1 \to S_2$ such that

$$f(\rho_1(m)(s)) = \rho_2(m)(f(s)) \text{ for all } m \in M \text{ and } s \in S_1.$$

If $f$ is injective, we say that the representation $\rho_2$ *extends* $\rho_1$.

**Remark 4.2.** (1) The above representations are called *boolean* because semilattices are precisely semimodules over the boolean semiring $2 = \{0, 1\}$ with $1 + 1 = 1$. For more on representations over general commutative semirings, see [21].

(2) The category of boolean representations of $M$ coincides with the functor category $\mathbf{JSL}_{\mathsf{f}}^{M}$, viewing $M$ as a one object category.

**Definition 4.3 (Canonical representation).** For every regular language $L$, the *canonical boolean representation* of the syntactic monoid $\mathsf{syn}(L)$ is given by

$$\kappa_L\colon \mathsf{syn}(L) \to \mathbf{JSL}(\mathsf{SLD}(L), \mathsf{SLD}(L)), \quad [w]_L \mapsto \lambda K.w^{-1}K.$$

It induces the *canonical boolean presentation* of the free monoid $\Sigma^*$ given by

$$\kappa_L \circ \mu_L\colon \Sigma^* \to \mathbf{JSL}(\mathsf{SLD}(L), \mathsf{SLD}(L)), \quad w \mapsto \lambda K.w^{-1}K,$$

where $\mu_L\colon \Sigma^* \twoheadrightarrow \mathsf{syn}(L)$ is the syntactic morphism.

The representation $\kappa_L \circ \mu_L$ amounts to constructing the transition semiring of the minimal **JSL**-automaton $\mathsf{SLD}(L)$, i.e. the *syntactic semiring* [35] of $L$.

**Example 4.4.** We describe the canonical boolean representation $\kappa_{L_n}$ for the language $L_n := (0+1)^*1(0+1)^n$, $n \in \mathbb{N}$. Let $S := 2_\perp^{n+1}$ be the semilattice of binary words of length $n+1$, ordered pointwise, with an additional bottom element $\perp$. Then $\mathsf{SLD}(L_n)$ is isomorphic to $S$, as witnessed by the isomorphism

$$f\colon S \xrightarrow{\cong} \mathsf{SLD}(L_n), \quad f(\perp) = \emptyset, \quad f(w) = w^{-1}L_n.$$

Thus, $\kappa_{L_n}$ is isomorphic to the representation $\rho\colon \mathsf{syn}(L_n) \to \mathbf{JSL}(S, S)$ where:

(1) $\rho([0]_{L_n})\colon S \to S$ performs a left-shift (distinct from left-rotate);

(2) $\rho([1]_{L_n})\colon S \to S$ performs a left-shift and sets the last bit as 1.

Finally, $\deg(\kappa_{L_n}) = \deg(\rho) = 1 + |J(2^{n+1})| = n+2$ is the number of states of the usual minimal nfa for $L$.

**Example 4.5.** We describe the canonical boolean presentation $\kappa_L$ for the language $L = a_1(a_2+a_3) + a_2(a_1+a_3) + a_3(a_1+a_2)$ over $\Sigma = \{a_1, a_2, a_3\}$. Consider the $\cup$-semilattice $M_3 = \{\emptyset, \{a_1,a_2\}, \{a_1,a_3\}, \{a_2,a_3\}, \Sigma\}$. Then $\mathsf{SLD}(L)$ is isomorphic to the product semilattice $2 \times M_3 \times 2$ via the map

$$f\colon \mathsf{SLD}(L) \xrightarrow{\cong} 2 \times M_3 \times 2, \quad f(X) = (X \cap \Sigma^2, X \cap \Sigma, X \cap \{\varepsilon\}).$$

Note that the first and third component is either $\emptyset$ or one other set, i.e. it may be identified with the elements of 2. For $i = 1,2,3$ we define the following semilattice morphisms:

$$\begin{aligned} &\alpha_i\colon 2 \to M_3, && \alpha_i(1) = \Sigma \setminus \{a_i\};\\ &\beta_i\colon M_3 \to 2, && \beta_i(S) = 1 \iff a_i \in S;\\ &\gamma\colon 2 \to 2 && \gamma(1) = 0;\\ &\delta\colon M_3 \times 2 \times 2 \to 2 \times M_3 \times 2, && \delta(x,y,z) = (z,x,y). \end{aligned}$$

Then $\kappa_L$ is isomorphic to $\rho\colon \mathsf{syn}(L) \to \mathbf{JSL}(2 \times M_3 \times 2,\ 2 \times M_3 \times 2)$ where

$$\rho([a_i]_L) = (\,2 \times M_3 \times 2 \xrightarrow{\alpha_i \times \beta_i \times \gamma} M_3 \times 2 \times 2 \xrightarrow{\delta} 2 \times M_3 \times 2\,).$$

Thus, $\deg(\kappa_L) = \deg(\rho) = 1+3+1 = 5$. An analogous description of $\kappa_L$ exists for any language $L$ where each word has the same length.

The next theorem links minimal nfas and representations.

**Definition 4.6.** The *nondeterministic state complexity* $\mathrm{ns}(L)$ of a regular language $L$ is the least number of states of any nfa accepting $L$.

**Theorem 4.7.** *For every regular language $L$, the nondeterministic state complexity* $\mathrm{ns}(L)$ *is the least degree of any boolean representation extending the canonical representation* $\kappa_L \circ \mu_L \colon \Sigma^* \to \mathbf{JSL}(\mathsf{SLD}(L), \mathsf{SLD}(L))$.

*Proof (Sketch).*

(1) Given a $k$-state nfa $N = (Q, \delta, I, F)$ accepting $L$, consider the subsemilattice $\mathsf{langs}(N) = \mathsf{simple}(\mathcal{P}(N))$ of $\mathcal{P}(\Sigma^*)$ on all languages accepted by subsets of $Q$. The embedding $\mathsf{SLD}(L) \rightarrowtail \mathsf{langs}(N)$ yields an extension of $\kappa_L \circ \mu_L$. Since the semilattice $\mathsf{langs}(N)$ is generated by the languages accepted by single states of $N$, this extension has degree at most $k$.

(2) Conversely, let $\rho\colon \Sigma^* \to \mathbf{JSL}(S, S)$ be a boolean representation of degree $k$ extending $\kappa_L \circ \mu_L$, witnessed by an injective equivariant map $h\colon \mathsf{SLD}(L) \rightarrowtail S$. One can equip $S$ with a **JSL**-dfa structure making $h$ an automata morphism. Since morphisms preserve accepted languages, it follows that $S$ accepts $L$. Then the nfa of join-irreducibles of $S$, see Remark 3.4, is a $k$-state nfa accepting $L$. □

As an application, let us return to the dependency relation $\mathcal{DR}_L$ introduced in Remark 3.14(2). Recall that a *biclique* of a relation $R \subseteq X \times Y$ (viewed as a bipartite graph) is a subset of the form $X' \times Y' \subseteq R$, where $X' \subseteq X$ and $Y' \subseteq Y$. A *biclique cover* of $R$ is a set $\mathscr{C}$ of bicliques with $R = \bigcup \mathscr{C}$. The *bipartite dimension* $\dim(R)$ is the least cardinality of any biclique cover of $R$.

**Theorem 4.8 (Gruber-Holzer [18]).** *For every regular language $L$, we have*

$$\dim(\mathcal{DR}_L) \leq \mathrm{ns}(L).$$

We give a new algebraic proof of this result based on boolean representations.

*Proof.* (1) The task of computing biclique covers is well-known to be equivalent to the *set basis* problem. Given a family $C \subseteq \mathcal{P}(Y)$ of subsets of a finite set $Y$, a set basis for $C$ is a family $B \subseteq \mathcal{P}(Y)$ such that each element of $C$ can be expressed as a union of elements of $B$. A relation $R \subseteq X \times Y$ has a biclique cover of size $k$ iff the family $C_R = \{R[x] : x \in X\} \subseteq \mathcal{P}(Y)$ of neighborhoods of nodes in $X$ has a set basis of size $k$.

(2) Given an instance $C \subseteq \mathcal{P}(Y)$ of the set basis problem, consider the $\cup$-subsemilattice $\langle C \rangle \subseteq \mathcal{P}(Y)$ generated by $C$, i.e. the semilattice of all unions of sets in $C$. We claim that $C$ has a set basis of size at most $k$ iff there exists an extension of $\langle C \rangle$ of degree at most $k$, i.e. a monomorphism $\langle C \rangle \rightarrowtail S$ into some finite semilattice $S$ with $|J(S)| \leq k$.

For the "only if" direction, suppose that $B \subseteq \mathcal{P}(Y)$ is a set basis of $C$ of size at most $k$. The the embedding $\langle C \rangle \rightarrowtail \langle B \rangle$ gives an extension of $\langle C \rangle$ with the desired property: since the semilattice $\langle B\rangle$ has a set of generators with at most $k$ elements, it has at most $k$ join-irreducibles.

For the "if" direction, suppose that $m\colon \langle C\rangle \rightarrowtail S$ with $|J(S)| \leq k$ is given. Since the free semilattice $\mathcal{P}(Y)$ is an injective object of **JSL** [19, Corollary 2.9], there exists a morphism $f\colon S \to \mathcal{P}(Y)$ extending the embedding $\langle C\rangle \rightarrowtail \mathcal{P}(Y)$. Consider the image $S' \subseteq \mathcal{P}(Y)$ of $f$, leading to the commutative diagram below:

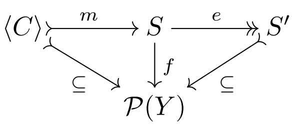

We thus have $\langle C\rangle \subseteq S' \subseteq \mathcal{P}(Y)$. Every set of generators of the semilattice $S'$ is a basis of $C$. Since the morphism $e$ is surjective, we have $|J(S')| \leq |J(S)| \leq k$, i.e. $S'$ has a set of generators with at most $k$ elements.

(3) Let $C_{\mathcal{DR}_L} \subseteq \mathcal{P}(\mathsf{LD}(L^{\mathsf{r}}))$ be the instance of the set basis problem corresponding to the dependency relation $\mathcal{DR}_L \subseteq \mathsf{LD}(L) \times \mathsf{LD}(L^{\mathsf{r}})$. Note that $\langle C_{\mathcal{DR}_L}\rangle$ consists of all $\mathcal{DR}_L[X]$ for $X \subseteq \mathsf{LD}(L)$. Thus, Theorem 3.15(1) shows that $\langle C_{\mathcal{DR}_L}\rangle \cong \mathsf{SLD}(L)$. In particular, every extension of the canonical boolean representation of $\Sigma^*$ yields an extension of the semilattice $\langle C_{\mathcal{DR}_L}\rangle$ of the same degree. Therefore, by part (1) and (2) and Theorem 4.7, we have $\dim(\mathcal{DR}_L) \leq \mathrm{ns}(L)$, as required.

Theorem 4.7 motivates the following definition, which can be considered the key concept of our paper:

**Definition 4.9.** The *nondeterministic syntactic complexity* $\mathrm{n}\mu(L)$ of a regular language $L$ is the least degree of any boolean representation of $\mathsf{syn}(L)$ extending the canonical boolean representation $\kappa_L\colon \mathsf{syn}(L) \to \mathbf{JSL}(\mathsf{SLD}(L), \mathsf{SLD}(L))$.

Just like the degrees of boolean representations of $\Sigma^*$ determine the state complexity of nfas, we will provide an automata-theoretic characterization of $\mathrm{n}\mu(L)$ in terms of *subatomic* nfas in Theorem 4.14 below.

**Definition 4.10.** An nfa accepting the language $L$ is called

(1) *atomic* if each state accepts a language from $\mathsf{BLD}(L)$, and

(2) *subatomic* if each state accepts a language from $\mathsf{BLRD}(L)$.

The notion of an atomic nfa goes back to Brzozowski and Tamm [6], as does the following characterization.

**Notation 4.11.** For any nfa $N$, let $\mathsf{rsc}(N)$ denote the dfa obtained via the *reachable subset construction*, i.e. the dfa-reachable part of $\mathcal{P}(N)$.

**Theorem 4.12.** *An nfa $N$ is atomic iff* $\mathsf{rsc}(N^{\mathsf{r}})$ *is a minimal dfa.*

We present a new conceptual proof, interpreting this theorem as an instance of the self-duality of **JSL**-dfas.

*Proof (Sketch).* Let $L$ be the language accepted by $N$. We establish the theorem by showing each of the following statements to be equivalent to the next one:

(1) $N$ is atomic.

(2) There exists a **JSL**-automata morphism from $\mathcal{P}(N)$ to $\mathsf{BLD}(L)$.

(3) There exists a **JSL**-automata morphism from $\mathcal{P}(\mathsf{dfa}(L^{\mathsf{r}}))$ to $\mathcal{P}(N^{\mathsf{r}})$.

(4) There exists a dfa morphism from $\mathsf{dfa}(L^{\mathsf{r}})$ to $\mathcal{P}(N^{\mathsf{r}})$.

(5) There exists a dfa morphism from $\mathsf{dfa}(L^{\mathsf{r}})$ to $\mathsf{rsc}(N^{\mathsf{r}})$.

(6) $\mathsf{rsc}(N^{\mathsf{r}})$ is a minimal dfa.

The key step is (2)⇔(3), which follows via duality from Lemmas 3.11 and 3.12, and Proposition 3.16. All remaining equivalences follow from the definitions. □

The next theorem gives an analogous characterization of subatomic nfas. Again, the proof is based on duality.

**Theorem 4.13.** *An nfa $N$ accepting the language $L$ is subatomic iff the transition monoid of* $\mathsf{rsc}(N^{\mathsf{r}})$ *is isomorphic to the syntactic monoid* $\mathsf{syn}(L^{\mathsf{r}})$.

*Proof (Sketch).* Each of the following statements is equivalent to the next one:

(1) $N$ is subatomic.

(2) There exists a **JSL**-dfa morphism from $\mathcal{P}(N)$ to $\mathsf{BLRD}(L)$.

(3) There exists a **JSL**-dfa morphism from $\mathsf{rdc}(\mathsf{simple}(P(N)))$ to $\mathsf{BLRD}(L)$.

(4) There exists a **JSL**-dfa morphism from $\mathcal{P}(\mathsf{syn}(L^{\mathsf{r}}))$ to $\mathsf{ts}(\mathsf{reach}(\mathcal{P}(N^{\mathsf{r}})))$.

(5) There exists a dfa morphism from $\mathsf{syn}(L^{\mathsf{r}})$ to $\mathsf{ts}(\mathsf{reach}(\mathcal{P}(N^{\mathsf{r}})))$.

(6) There exists a dfa morphism from $\mathsf{syn}(L^{\mathsf{r}})$ to $\mathsf{tm}(\mathsf{rsc}(N^{\mathsf{r}}))$.

(7) The monoids $\mathsf{syn}(L^{\mathsf{r}})$ and $\mathsf{tm}(\mathsf{rsc}(N^{\mathsf{r}}))$ are isomorphic.

The equivalence (3)⇔(4) follows via duality from Lemma 3.11, Proposition 3.17 and Proposition 3.19. All remaining equivalences follow from the definitions. □

We are prepared to state the main result of our paper, an automata-theoretic characterization of the nondeterministic syntactic complexity:

**Theorem 4.14.** *For every regular language $L$, the nondeterministic syntactic complexity* $\mathrm{n}\mu(L)$ *is the least number of states of any subatomic nfa accepting $L$.*

*Proof (Sketch).*

(1) Let $N$ be a $k$-state subatomic nfa accepting the language $L$. As in the proof of Theorem 4.7, we consider the semilattice $\mathsf{langs}(N) = \mathsf{simple}(\mathcal{P}(N))$. Then

$$\rho\colon \mathsf{syn}(L) \to \mathbf{JSL}(\mathsf{langs}(N), \mathsf{langs}(N)), \quad [w]_L \mapsto \lambda K.w^{-1}K,$$

is a representation of $\mathsf{syn}(L)$ of degree at most $k$ extending $\kappa_L$.

(2) Conversely, let $\rho\colon \mathsf{syn}(L) \to \mathbf{JSL}(S,S)$ be a boolean representation extending $\kappa_L$, and let $h\colon \mathsf{SLD}(Q) \rightarrowtail S$ be the embedding. As in the proof of Theorem 4.7, we can equip $S$ with the structure of a **JSL**-dfa making $h$ an automata morphism. Its nfa of join-irreducibles, see Remark 3.4, is a subatomic nfa accepting $L$ with $\deg(\rho)$ states. □

We conclude this section with the observation that the state complexity of unrestricted nfas, subatomic nfas and atomic nfas generally differs:

**Example 4.15 (Subatomic more succinct than atomic).** Consider the language $L$ accepted by the nfa $N$ shown below, along with the minimal dfas for $L$ and $L^{\mathsf{r}}$. Each automaton has exactly one initial state, namely 0.

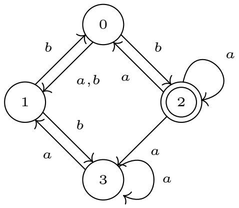

$N$

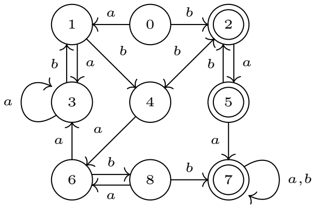

$\mathsf{dfa}(L)$

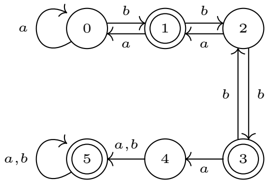

$\mathsf{dfa}(L^{\mathsf{r}})$

Brzozowski and Tamm [6] showed that there is no atomic nfa with four states accepting $L$. However, $N$ is subatomic: one can verify that the transition monoids of $\mathsf{dfa}(L^{\mathsf{r}})$ and $\mathsf{rsc}(N^{\mathsf{r}})$ both have 22 elements. Since the former is the syntactic monoid of $L^{\mathsf{r}}$, they are isomorphic, and so Theorem 4.13 applies.

**Example 4.16 (Subatomic less succinct than general nfas).** There is a regular language for which no state-minimal nfa is subatomic:

$$L := \{ a^n : n \in \mathbb{N}, n \neq 5 \} \subseteq \{a\}^*.$$

It is accepted by the following nfa:

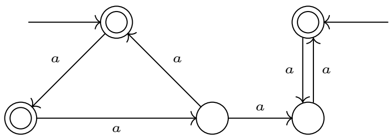

An exhaustive search shows that no subatomic nfa with five states accepts $L$. In fact, $L$ is the unique (!) unary language with $\mathrm{ns}(L) \leq 5$ and $\mathrm{ns}(L) < \mathrm{n}\mu(L)$. Moreover, the above nfa and its reverse are the only state-minimal nfas for $L$.

## 5 Applications

While subatomic nfas are generally less succinct then unrestricted ones, all structural results concerning nondeterministic state complexity we have encountered in the literature are actually about nondeterministic syntactic complexity: they implicitly identify classes of languages where the two measures coincide. In the present section, we illustrate this in a few selected applications.

### 5.1 Unary languages

For unary languages $L \subseteq \{a\}^*$, two-sided derivatives are left derivatives. Thus, a unary nfa is atomic iff it is subatomic.

**Example 5.1 (Cyclic unary languages).** A unary language $L$ is *cyclic* if its minimal dfa is a cycle [16]. We claim that $\mathrm{ns}(L) = \mathrm{n}\mu(L)$. To see this, let $d := |\mathsf{LD}(L)|$ be the *period* (i.e. number of states) of the minimal dfa. By Fact 1 of [16] (originally from [22]) every state-minimal nfa $N$ accepting $L$ is a disjoint union of cyclic dfas whose periods divide $d$.[1] Then $|\mathsf{rsc}(N^{\mathsf{r}})| = d$: we have $|\mathsf{rsc}(N^{\mathsf{r}})| \geq d$ since $\mathsf{rsc}(N^{\mathsf{r}})$ is a dfa accepting $L = L^{\mathsf{r}}$ and $d$ is the size of the minimal dfa for $L$, and $|\mathsf{rsc}(N^{\mathsf{r}})| \leq d$ because after $d$ steps, each cycle will be back in its initial state. Thus $N$ is atomic by Theorem 4.12 and hence subatomic.

We deduce the following result for (not necessarily unary) regular languages:

**Theorem 5.2.** *If* $\mathsf{syn}(L)$ *is a cyclic group, then* $\mathrm{ns}(L) = \mathrm{n}\mu(L)$.

*Proof (Sketch).* Suppose that $\mathsf{syn}(L) = \mathsf{tm}(\mathsf{dfa}(L))$ is cyclic. Then there exists $w_0 \in \Sigma^*$ such that the map $\lambda X.w_0^{-1}X\colon \mathsf{LD}(L) \to \mathsf{LD}(L)$ generates $\mathsf{tm}(\mathsf{dfa}(L))$. Fix an alphabet $\Sigma_0 = \{a_0\}$ disjoint from $\Sigma$ and consider the unary language

$$L_0 := \{\, a_0^n \;:\; n \in \mathbb{N},\, w_0^n \in L \,\} \subseteq \Sigma_0^*.$$

Let $g : \Sigma_0^* \to \Sigma^*$ be the monoid morphism where $g(a_0) := w_0$. Then we have the **JSL**-isomorphism

$$f\colon \mathsf{SLD}(L_0) \xrightarrow{\cong} \mathsf{SLD}(L), \quad f(X^{-1}L_0) := [g[X]]^{-1}L.$$

For each $a \in \Sigma$ choose $n_a \in \mathbb{N}$ such that $a^{-1}K = (w_0^{n_a})^{-1}K$ for all $K \in \mathsf{LD}(L)$. The respective transition endomorphisms of the **JSL**-automata $\mathsf{SLD}(L_0)$ and $\mathsf{SLD}(L)$ determine each other in the sense that the following diagrams commute:

$$\begin{array}{ccc} \mathsf{SLD}(L_0) & \xrightarrow[\cong]{f} & \mathsf{SLD}(L) \\ {\scriptstyle a_0^{-1}(-)}\downarrow & & \downarrow{\scriptstyle w_0^{-1}(-)} \\ \mathsf{SLD}(L_0) & \xrightarrow[f]{\cong} & \mathsf{SLD}(L) \end{array} \qquad \begin{array}{ccc} \mathsf{SLD}(L_0) & \xrightarrow[\cong]{f} & \mathsf{SLD}(L) \\ {\scriptstyle (a_0^{n_a})^{-1}(-)}\downarrow & & \downarrow{\scriptstyle a^{-1}(-)} \\ \mathsf{SLD}(L_0) & \xrightarrow[f]{\cong} & \mathsf{SLD}(L) \end{array}$$

Then $\mathrm{ns}(L) = \mathrm{ns}(L_0)$ by Theorem 4.7 and $\mathrm{n}\mu(L) = \mathrm{n}\mu(L_0)$ by Theorem 4.14. Moreover, by Example 5.1 we know that $\mathrm{ns}(L_0) = \mathrm{n}\mu(L_0)$, so the claim follows.

**Example 5.3 ($\mathrm{n}\mu(L)$ no larger than Chrobak normal form).** A unary nfa is in *Chrobak normal form* [8,13] if it has a single initial state and at most one state with multiple successors, all of which lie in disjoint cycles. We claim that for any nfa $N$ in Chrobak normal form accepting the language $L$, we have

$$\mathrm{n}\mu(L) \leq |N|,$$

[1] In [16] nfas are restricted to have a single initial state and so are distinguished from unions of dfas; the latter are valid nfas from our perspective.

where $|N|$ denotes the number of states of $N$. To see this, observe that each state of $N$ up to and including the unique choice state accepts some left derivative of $L$. The successors of the choice state collectively accept a derivative $u^{-1}L$; this language is cyclic because it is a finite union of cyclic languages. Therefore, by Example 5.1 we may replace the cycles by an atomic nfa accepting $u^{-1}L$, without increasing the number of states. The resulting nfa is atomic.

Since every unary nfa on $n$ states can be transformed into an nfa in Chrobak normal form with $O(n^2)$ states [8, Lemma 4.3], we get:

**Corollary 5.4.** *If $L$ is a unary regular language, then* $\mathrm{n}\mu(L) = O(\mathrm{ns}(L)^2)$.

## 5.2 Languages with a canonical state-minimal nfa

There are several natural classes of regular languages for which *canonical* state-minimal nondeterministic acceptors have been identified. We show that these acceptors are actually subatomic. In our arguments, we frequently consider the *length* of a finite semilattice $S$, i.e. the maximum length $n$ of any ascending chain $s_0 < s_1 < \ldots < s_n$ in $S$. Note that since every element is uniquely determined by the set of join-irreducibles below it, the length of $S$ is at most $|J(S)|$.

**Example 5.5 (Bideterministic and biseparable languages).**

(1) A language is called *bideterministic* if it is accepted by a dfa whose reverse is also a dfa. In this case, the minimal dfa is a minimal nfa [34, 38]. Bideterministic languages have been studied in the context of automata learning [2] and coding theory, where they are known as *rectangular codes* [27, 36]. We show that for every bideterministic language $L$,

$$\mathrm{ns}(L) = \mathrm{n}\mu(L) = |\mathsf{LD}(L)|.$$

To this end, we first note that by [36, Theorem 3.1] a language $L \subseteq \Sigma^*$ is bideterministic iff the left derivatives of $L$ are pairwise disjoint. This implies that $\mathsf{SLD}(L)$ is a boolean algebra with atoms $\mathsf{LD}(L)$. Since the length of a boolean algebra equals the number of atoms (= join-irreducibles), we conclude that for every finite semilattice extension $\mathsf{SLD}(L) \rightarrowtail S$, the semilattice $S$ has length at least $|\mathsf{LD}(L)|$. Thus, $|\mathsf{LD}(L)| \leq |J(S)|$, so any representation $\rho$ extending $\kappa_L$ or $\kappa_L \circ \mu_L$ satisfies $|\mathsf{LD}(L)| \leq \deg(\rho)$. Hence, $\mathrm{ns}(L) = \mathrm{n}\mu(L) = |\mathsf{LD}(L)|$ by Theorem 4.7 and 4.14. In particular, the minimal dfa of $L$ is a minimal nfa.

(2) A language $L$ is *biseparable* if $\mathsf{SLD}(L)$ is a boolean algebra [28].[2] For every biseparable language $L$, the *canonical residual automaton* [12], i.e. the nfa $N_L$ of join-irreducibles of the minimal **JSL**-dfa $\mathsf{SLD}(L)$, is a state-minimal nfa; it is subatomic because every state of $N_L$ accepts a derivative of $L$. This follows exactly as in (1): our argument only used that $\mathsf{SLD}(L)$ is a boolean algebra.

[2] Actually [28] defines biseparability as a property of nfas, and characterizes biseparable nfas as those accepting a language $L$ for which no $\cup$-irreducible left derivative is contained in the union of other $\cup$-irreducible left derivatives. This is equivalent to the lattice $\mathsf{SLD}(L)$ being boolean, i.e. to $L$ being 'biseparable' in our sense.

**Example 5.6 (Maximal reachability).** A folklore result asserts that if $N$ is an nfa whose accepted language $L$ satisfies $|\mathsf{LD}(L)| = 2^{|N|}$, then $N$ is state-minimal. Since $\mathsf{LD}(L)$ forms the set of states of the minimal dfa for $L$ and $\mathsf{rsc}(N)$ accepts $L$, we have $\mathsf{rsc}(N) = \mathcal{P}(N)$. It follows the **JSL**-dfa $\mathcal{P}(N)$ is reachable and simple, hence isomorphic to the minimal **JSL**-dfa $\mathsf{SLD}(L)$. This proves that $\mathsf{SLD}(L)$ is a boolean algebra, i.e. $L$ is a biseparable language. We conclude from Example 5.5(2) that $\mathrm{ns}(L) = \mathrm{n}\mu(L) = |N|$ and $N_L$ is a subatomic minimal nfa.

**Example 5.7 (BiRFSA and topological languages).** So far $\mathsf{SLD}(L)$ has been a boolean algebra. But the argument in Example 5.5 also applies when $\mathsf{SLD}(L)$ is a distributive lattice, noting that the length of a finite distributive lattice is equal to the number of its join-irreducibles [17, Corollary 2.14]. Languages with this property are called *topological* [1]. It thus follows as in Example 5.5(2) that for any topological language $L$, the canonical residual automaton $N_L$ is subatomic and a state-minimal nfa. Thus, $\mathrm{ns}(L) = \mathrm{n}\mu(L) = |J(\mathsf{SLD}(L))|$.

There is another class of languages where $N_L$ is known to be a state-minimal nfa, the *biRFSA* languages [28]. A language $L$ is called biRFSA if $N_L$ is isomorphic to $(N_{L^\mathsf{r}})^\mathsf{r}$. Surprisingly, these languages are exactly the topological ones:

(1) *Suppose that L is topological.* Recall that $N_L$ is the nfa of join-irreducibles of the minimal **JSL**-dfa. Thus, it has states $J(\mathsf{SLD}(L))$ and transitions given by $X \xrightarrow{a} Y$ iff $Y \subseteq a^{-1}X$ for $a \in \Sigma$. Moreover, a join-irreducible $j$ is initial iff $j \subseteq L$ and final iff $\varepsilon \in j$. Since the lattice $\mathsf{SLD}(L)$ is distributive, we have a canonical bijection between its join- and meet-irreducibles:

$$\tau\colon J(\mathsf{SLD}(L)) \xrightarrow{\cong} M(\mathsf{SLD}(L)), \quad \tau(j) = \bigcup\{X \in \mathsf{SLD}(L) : j \nsubseteq X\}.$$

Let $\theta$ be the unique map making the following diagram commute, where $\mathrm{dr}_L$ is the restriction of the isomorphism of Proposition 3.13:

$$\begin{array}{ccc} & J(\mathsf{SLD}(L)) & \\ {}^{\theta}\swarrow\cong & & \cong\searrow^{\tau} \\ J(\mathsf{SLD}(L^\mathsf{r})) & \xrightarrow[\mathrm{dr}_L]{\cong} & M(\mathsf{SLD}(L)) \end{array}$$

One can show $\theta$ to be an nfa isomorphism from $N_L$ to $(N_{L^\mathsf{r}})^\mathsf{r}$. Thus, $L$ is biRFSA.

(2) *Suppose that L is biRFSA.* Then we have a surjective **JSL**-morphism

$$[\mathcal{P}(J(\mathsf{SLD}(L)))]^{\mathsf{op}} \cong \mathcal{P}(J(\mathsf{SLD}(L^\mathsf{r}))) \xrightarrow{e_{L^\mathsf{r}}} \mathsf{SLD}(L^\mathsf{r}) \cong [\mathsf{SLD}(L)]^{\mathsf{op}},$$

where the first isomorphism follows from $N_L \cong (N_{L^\mathsf{r}})^\mathsf{r}$ and Lemma 3.11, the second isomorphism is given by Proposition 3.13, and $e_{L^\mathsf{r}}$ sends $X \subseteq J(\mathsf{SLD}(L^\mathsf{r}))$ to $\bigcup X$. The dual of this morphism is the injective **JSL**-morphism

$$m_L\colon \mathsf{SLD}(L) \rightarrowtail \mathcal{P}(J(\mathsf{SLD}(L)))$$

sending $K \in \mathsf{SLD}(L)$ to the set of all $j \in J(\mathsf{SLD}(L))$ with $j \subseteq K$. Note that $e_L \circ m_L = id_{\mathsf{SLD}(Q)}$, showing that $\mathsf{SLD}(L)$ is a retract of $\mathcal{P}(J(\mathsf{SLD}(L)))$. Since **JSL**-retracts of finite distributive lattices are distributive, see e.g. [31, Lemma 2.2.3.15], it follows that $\mathsf{SLD}(L)$ is distributive. Thus, $L$ is topological.

**Example 5.8 (Extremal languages).** Call a language *extremal* if $\mathsf{SLD}(L)$ has length $|J(\mathsf{SLD}(L))|$ i.e. we have an *extremal lattice* in the sense of Markowsky [29]. Again, the argument of Example 5.5 applies and we get $\mathrm{ns}(L) = \mathrm{n}\mu(L) = |J(\mathsf{SLD}(L))|$. Topological languages are extremal since every distributive lattice is an extremal lattice, although extremal languages need not be topological. Both classes are naturally characterized in terms of the reduced dependency relation:

(1) $L$ is topological iff $\mathcal{DR}_L^j$ is essentially an order relation $\leq_P \subseteq P \times P$ of a finite poset [30, Example 2.2.12].

(2) $L$ is extremal iff $\mathcal{DR}_L^j$ is *upper unitriangularizable* [29, Theorem 11].

The latter means the adjacency matrix of the bipartite graph $\mathcal{DR}_L^j$ can be put in upper triangular form with ones along the diagonal, by permuting rows and columns. An order relation is upper unitriangularizable because it may be extended to a linear order.

## 6 Conclusion and Future Work

Motivated by the duality theory of deterministic finite automata over semilattices, we introduced a natural class of nondeterministic finite automata called *subatomic nfas* and studied their state complexity in terms of boolean representations of syntactic monoids. Furthermore, we demonstrated that a large body of previous work on state minimization of general nfas actually constructs minimal subatomic ones. There are several directions for future work.

As illustrated by Theorem 4.8, the dependency relation $\mathcal{DR}_L$ forms a useful tool for proving lower bounds on nfas. It is also a key element of the Kameda-Weiner algorithm [26, 37] for minimizing nfas, which rests on computing biclique covers of $\mathcal{DR}_L$. We aim to give an algebraic interpretation of dependency relations based on the representation of finite semilattices by contexts [24], which can be augmented to a categorical equivalence between $\mathbf{JSL}_\mathsf{f}$ and a suitable category of bipartite graphs [31]. Under this equivalence, **JSL**-dfas correspond to *dependency automata*; in particular, the minimal **JSL**-dfa $\mathsf{SLD}(L)$ corresponds to a dependency automaton whose underlying bipartite graph is precisely the dependency relation $\mathcal{DR}_L$. We expect that this observation can lead to a fresh algebraic perspective on the Kameda-Weiner algorithm, as well as a generalization of it computing minimal (sub-)atomic nfas.

On a related note, we also intend to investigate the complexity of the minimization problem for (sub-)atomic nfas. While minimizing general nfas is PSPACE-complete, even if the input automaton is a dfa, we conjecture that the additional structure present in (sub-)atomic acceptors will simplify their minimization to an NP-complete task. First evidence in this direction is provided by Geldenhuys, van der Merve, and van Zijl [14] whose work implies that minimal atomic nfas can be efficiently computed in practice using SAT solvers.

## References

[1] Adámek, J., Myers, R.S., Urbat, H., Milius, S.: On continuous nondeterminism and state minimality. In: Proc. 30th Conference on the Mathematical Foundations of Programming Semantics (MFPS XXX). vol. 308, pp. 3–23 (2014)

[2] Angluin, D.: Inference of reversible languages. J. ACM **29**(3), 741–765 (1982)

[3] Arbib, M.A., Manes, E.G.: Adjoint machines, state-behavior machines, and duality. Journal of Pure and Applied Algebra **6**(3), 313–344 (1975)

[4] Arbib, M.A., Manes, E.G.: Fuzzy machines in a category. Bulletin of the Australian Mathematical Society **13**(2), 169–210 (1975)

[5] Backhouse, R.: Factor theory and the unity of opposites. Journal of Logical and Algebraic Methods in Programming **85**(5, Part 2), 824–846 (2016)

[6] Brzozowski, J., Tamm, H.: Theory of átomata. Theoretical Computer Science **539**, 13–27 (2014)

[7] Brzozowski, J.A.: Derivatives of regular expressions. J. ACM **11**(4), 481–494 (Oct 1964)

[8] Chrobak, M.: Finite automata and unary languages. Theoretical Computer Science **47**, 149–158 (1986)

[9] Clemente, L., Mayr, R.: Efficient reduction of nondeterministic automata with application to language inclusion testing. Logical Methods in Computer Science **Volume 15, Issue 1** (2019)

[10] Conway, J.H.: Regular Algebra and Finite Machines. Printed in GB by William Clowes & Sons Ltd (1971)

[11] De Wulf, M., Doyen, L., Henzinger, T.A., Raskin, J.F.: Antichains: A new algorithm for checking universality of finite automata. In: Ball, T., Jones, R.B. (eds.) Computer Aided Verification. pp. 17–30. Springer (2006)

[12] Denis, F., Lemay, A., Terlutte, A.: Residual finite state automata. In: Ferreira, A., Reichel, H. (eds.) STACS 2001: 18th Annual Symposium on Theoretical Aspects of Computer Science Dresden, Germany, February 15–17, 2001 Proceedings. pp. 144–157. Springer Berlin Heidelberg, Berlin, Heidelberg (2001)

[13] Gawrychowski, P.: Chrobak normal form revisited, with applications. In: Bouchou-Markhoff, B., Caron, P., Champarnaud, J.M., Maurel, D. (eds.) Implementation and Application of Automata. pp. 142–153. Springer Berlin Heidelberg, Berlin, Heidelberg (2011)

[14] Geldenhuys, J., van der Merwe, B., van Zijl, L.: Reducing nondeterministic finite automata with SAT solvers. In: Yli-Jyrä, A., Kornai, A., Sakarovitch, J., Watson, B. (eds.) Finite-State Methods and Natural Language Processing. pp. 81–92. Springer Berlin Heidelberg, Berlin, Heidelberg (2010)

[15] Goguen, J.A.: Discrete-time machines in closed monoidal categories. I. J. Comput. Syst. Sci. **10**(1), 1–43 (1975)

[16] Gramlich, G.: Probabilistic and nondeterministic unary automata. In: Proc. of Math. Foundations of Computer Science, Springer, LNCS 2747, 2003. pp.460–469. Springer (2003)

[17] Grätzer, G.: General Lattice Theory. Birkhäuser Verlag, 2. edn. (1998)

[18] Gruber, H., Holzer, M.: Finding lower bounds for nondeterministic state complexity is hard. In: Ibarra, O.H., Dang, Z. (eds.) Developments in Language Theory: 10th International Conference, DLT 2006, Santa Barbara, CA, USA, June 26-29, 2006. Proceedings. pp. 363–374. Springer Berlin Heidelberg, Berlin, Heidelberg (2006)

[19] Horn, A., Kimura, N.: The category of semilattices. Algebra Univ. **1**, 26–38 (1971)

[20] Hromkovič, J., Seibert, S., Karhumäki, J., Klauck, H., Schnitger, G.: Communication complexity method for measuring nondeterminism in finite automata. Information and Computation **172**(2), 202–217 (2002), http://www.sciencedirect.com/science/article/pii/S089054010193069X

[21] Izhakian, Z., Rhodes, J., Steinberg, B.: Representation theory of finite semigroups over semirings. Journal of Algebra **336**(1), 139–157 (2011)

[22] Jiang, T., McDowell, E., Ravikumar, B.: The structure and complexity of minimal nfa’s over a unary alphabet. International Journal of Foundations of Computer Science **02**(02), 163–182 (1991)

[23] Jiang, T., Ravikumar, B.: Minimal NFA problems are hard. SIAM Journal on Computing **22**(6), 1117–1141 (1993)

[24] Jipsen, P.: Categories of algebraic contexts equivalent to idempotent semirings and domain semirings. In: Kahl, W., Griffin, T.G. (eds.) Relational and Algebraic Methods in Computer Science. pp. 195–206. Springer Berlin Heidelberg, Berlin, Heidelberg (2012)

[25] Johnstone, P.T.: Stone spaces. Cambridge University Press (1982)

[26] Kameda, T., Weiner, P.: On the state minimization of nondeterministic finite automata. IEEE Transactions on Computers **C-19**(7), 617–627 (1970)

[27] Kschischang, F.R.: The trellis structure of maximal fixed-cost codes. IEEE Transactions on Information Theory **42**(6), 1828–1838 (1996)

[28] Latteux, M., Roos, Y., Terlutte, A.: Minimal NFA and biRFSA languages. RAIRO - Theoretical Informatics and Applications **43**(2), 221–237 (2009)

[29] Markowsky, G.: Primes, irreducibles and extremal lattices. Order **9**, 265–290 (09 1992)

[30] Myers, R.S.R.: Nondeterministic automata and JSL-dfas. CoRR **abs/2007.06031** (2020), https://arxiv.org/abs/2007.06031

[31] Myers, R.S.R.: Representing semilattices as relations. CoRR **abs/2007.10277** (2020), https://arxiv.org/abs/2007.10277

[32] Myers, R.S.R., Adámek, J., Milius, S., Urbat, H.: Coalgebraic constructions of canonical nondeterministic automata. Theoretical Computer Science **604**, 81–101 (2015)

[33] Pin, J.É.: Mathematical foundations of automata theory (September 2020), available at http://www.liafa.jussieu.fr/~jep/PDF/MPRI/MPRI.pdf

[34] Pin, J.E.: On reversible automata. In: Simon, I. (ed.) LATIN ’92. pp. 401–416. Springer Berlin Heidelberg, Berlin, Heidelberg (1992)

[35] Polák, L.: Syntactic semiring of a language. In: Sgall, J., Pultr, A., Kolman, P. (eds.) Mathematical Foundations of Computer Science 2001: 26thInternational Symposium, MFCS 2001 Mariánské Lázne, Czech Republic, August 27–31, 2001 Proceedings. pp. 611–620. Springer Berlin Heidelberg, Berlin, Heidelberg (2001)

[36] Shankar, P., Dasgupta, A., Deshmukh, K., Rajan, B.: On viewing block codes as finite automata. Theoretical Computer Science **290**(3), 1775–1797 (2003)

[37] Tamm, H.: New interpretation and generalization of the Kameda-Weiner method. In: Chatzigiannakis, I., Mitzenmacher, M., Rabani, Y., Sangiorgi, D. (eds.) ICALP 2016, Rome, Italy. LIPIcs, vol. 55, pp. 116:1–116:12. SchlossDagstuhl - Leibniz-Zentrum für Informatik (2016)

[38] Tamm, H., Ukkonen, E.: Bideterministic automata and minimal representations of regular languages. Theoretical Computer Science **328**(1), 135–149 (2004)

[39] van Heerdt, G., Moerman, J., Sammartino, M., Silva, A.: A (co)algebraic theory of succinct automata. Journal of Logical and Algebraic Methods in Programming **105**, 112–125 (2019)

